# Influence of autophagy on the efficacy of radiotherapy

**DOI:** 10.1186/s13014-017-0795-y

**Published:** 2017-03-21

**Authors:** Shing Yau Tam, Vincent Wing Cheung Wu, Helen Ka Wai Law

**Affiliations:** Department of Health Technology and Informatics, Faculty of Health and Social Sciences, The Hong Kong Polytechnic University, Hong Kong, China

**Keywords:** Autophagy, Radiotherapy, Signalling pathway, Radiotherapy efficacy, Radiosensitivity, Cancer Cell lines

## Abstract

Autophagy is an important catabolic process in which cells digest and recycle their own cytoplasmic contents for maintaining cellular homeostasis. Interestingly, autophagy could play both pro-death and pro-survival roles in influencing the development of cancer via various signal pathways. As radiotherapy is one of the main treatment modalities for cancer, we reviewed the effect of autophagy modulations on radiosensitivity and radiotherapy efficacy in various cancer types. The future development of autophagy modifications for improving radiotherapy efficacy and cancer prognosis will also be discussed.

## Background

**Fig. 1:**
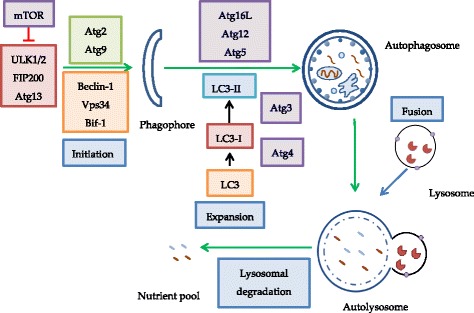
Title: An overview of the central autophagy mechanism. The ULK and Beclin 1 protein complexes initiate phagophore formation. Atg5/12/16 and LC3-II are responsible for expansion to autophagosome. Fusion of autophagosome and lysosome produces autolysosome as the final process. Legend: Green arrows represent activating processes for autophagy stimulation and the red arrow represents a repressing process for autophagy inhibition

Autophagy, a word derived from Greek “auto” (self) and “phagos” (to eat), is a catabolic process in which the cells digest and recycle their own cytoplasmic contents. This critical process is evolutionarily conserved from unicellular organisms to humans and continuously occurs at basal level to ensure healthy cellular homeostasis by eliminating waste and long-lived or damaged cellular constituents. There are three types of autophagy, including macroautophagy, microautophagy and chaperone-mediated autophagy [1]. Macroautophagy (hereby referred to as autophagy) is the major autophagy pathway to be discussed in this review. The process is controlled by 36 highly conserved genes which are known as AuTophaGy genes (ATGs) and starts with double-membrane vesicles called autophagosomes, which would fuse with lysosomes to degrade cytoplasmic contents back to their original constituents by hydrolytic enzymes (Fig. 1). Different stimuli including aggregated or misfolded proteins, stress, pathogens, cytokines, starvation and protein synthesis inhibition might induce autophagy. Apart from maintaining cellular homeostasis, autophagy (or autophagy defects), may lead to several pathological conditions, including cancer [2–4].

## Relation of autophagy to cancer

Autophagy plays an important role in cancer because of its tumour suppressing and tumour protecting function. For tumour suppressing function at the initiation stage, ATG Beclin-1 (Fig. 1) was identified as a tumour suppressor gene as it is mono-allelically deleted in many cases including ovarian cancers (75%), breast cancers (50–70%) and prostate cancers (40%) [5]. Also, Beclin-1 is allelically deleted and weakly expressed in most human breast carcinoma cell lines while the normal epithelium cells demonstrated a much higher expression [6]. In addition, overexpression of Beclin-1 in human breast carcinoma cell line MCF-7 cells could reduce tumourigenesis by inhibiting cell proliferation in a xenograft model [2]. Thus, low expression of Beclin-1 could favour the development of cancer. For colorectal and gastric cancers, associations were found with the down-regulation of Bif-1 and Atg2B, Atg5, Atg9B and Atg12 mutations, which led to inhibition of programmed cell death in colon cancer (Fig. 1). Also, mutation of exon 8 of UV radiation resistance-associated gene (UVRAG) reduced autophagy and promoted these cancer types [2, 4]. Taken together, there is broad evidence that autophagy and ATG type of proteins have a tumour suppressive role and down-regulation of the latter can promote tumourigenesis in early stage tumours.

Apart from direct suppression of tumourigenesis, autophagy could also induce cellular senescence, which is a state of stable cell cycle arrest that protect the cells against a variety of cellular insults. It is a delayed stress response with multiple effector mechanisms including oncogene-induced senescence. Oncogene initially induces a highly proliferative state in cells. However, senescence will gradually replace the mitotic burst state and this illustrates its tumour suppressing role. A past study has demonstrated that the autophagy marker microtubule-associated protein 1 light chain 3β (MAP1LC3) was up-regulated in Ras oncogene-induced senescence and resulted in the accumulation of autophagosomes in these cells [7]. The lack of Atg5 or Atg7 could also diminish oncogene-induced senescence and delay the production of senescence-associated cytokine [4]. These pieces of evidence support the notion that senescence may be promoted by autophagy and basal autophagy is important in restricting proliferation during oncogenic stress. In addition, combining autophagy activation by rapamycin and irradiation could initiate premature senescence in both in vitro and in vivo models of radiation-resistant glioblastoma and parotid carcinoma cells. In the in vitro model, irradiation increases autophagic flux for 72 h and the addition of rapamycin further intensified the effect. During this period, the senescence-associated β-galactosidase activity also showed that premature senescence has been initiated, caused by the inhibition of mechanistic target of rapamycin (mTOR) pathway. Similar results have also been obtained in tumour xenografts [8].

In a converse way to the pro-death roles of autophagy in cancer cells, autophagy also has pro-survival functions. As vascularisation is poor in fast growing and late stage tumours with populations of tumour cells beyond oxygen diffuse distance or supported by malformed vessels, autophagy is stimulated by an inadequate oxygen and nutrient supply and inefficient waste removal. In addition, the withdrawal of growth factors, accumulation of oxidised and aggregated proteins and intracellular calcium, production of reactive oxygen species (ROS) and ammonia might also stimulate autophagy [2]. In these stressful conditions, autophagy helps tumour cells to maintain homeostasis and becomes a main determent of successful therapy, including radiotherapy. However, excessive stress could also lead to cell death due to the degeneration of the majority of cellular contents [9]. In this review, the role of autophagy in influencing radiosensitivity of various cancer types will be discussed in detail.

## Relation of autophagy with ionising radiation

Numerous pharmaceutical studies have tried to promote or inhibit autophagy through various pathways in order to improve the outcome of cancer treatment. However, the relationship between radiotherapy and autophagy has not been studied in depth. As apoptosis only accounts for 20% or less of radiation-induced cell death, other cell death pathways including autophagy should also be studied [10]. It is known that radiotherapy treatment is one of the stresses that induces autophagy in both cancer and normal cells [11]. An in vitro study involving the irradiation of glioblastoma multiforme (GBM) cells has shown that cells have died through autophagy without the involvement of apoptosis [12]. Although the specific mechanism that links between radiation and autophagy has not been well established, there were some studies that linked mTOR pathway and endoplasmic reticulum (ER) stress to radiation-induced cell death. For the mTOR pathway, radiation could cause decreased phosphorylation of the autophosphorylation site of p-mTOR (decreased p-mTOR/mTOR ratio) in an in vitro study of MCF-7 breast cancer cell line [13]. While for ER stress, radiation could cause increased protein kinase-like endoplasmic reticulum kinase (PERK)/ eukaryotic initiation factor 2α (eIF2α) expression in caspase-3/7-deficient cells or ER stressor tunicamycin (TM) treated MCF-7 cells, and increased endoplasmic reticulum protein 29 (ERp29) expression in intestinal epithelial cells IEC-6 [14, 15]. The radiation-induced ER stress protein expression is linked to protein folding and unfolded protein response (UPR) and thus, radiation-induced autophagy [10].

## Ionising radiation-related autophagy signalling pathways

### PI3K-Akt-mTOR

**Fig. 2:**
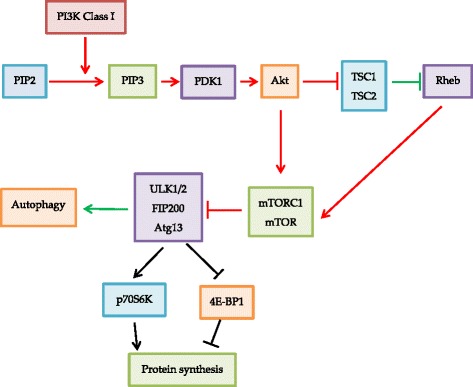
Title: In PI3K-Akt-mTOR signaling pathway, PI3K inhibits autophagy via activation of Akt. Akt promotes mTORC1 both directly and indirectly through inhibiting TSC complexes. mTORC1 directly inhibits autophagy as the final step. Legend: Green arrows represent activating or repressing processes for autophagy stimulation and red arrows represent activating or repressing processes for autophagy inhibition

The phosphatidylinositol-3-kinase (PI3K)-protein kinase B (Akt)-mTOR pathway (Fig. 2) is one of the most important autophagy signalling pathways in cancer growth and progression [2]. Hormones, growth factors, tumour suppressors and oncogenes can activate class-I PI3K to catalyse phosphatidylinositol-3 phosphate production, causing activation and phosphorylation of a serine/threonine kinase PI3K-Akt. Akt will then activate mTOR through ribosomal protein S6 kinase (RPS6KB1) and by phosphorylating and inhibiting tuberous sclerosis complex (TSC), which originally inhibits mTOR [2]. mTOR is a part of two multiprotein complexes, namely mechanistic target of rapamycin complex 1 and 2 (mTORC1 and mTORC2). In addition, Ras homolog enriched in brain (Rheb-GTP) can act as a substrate for TSC2 and activate mTORC1 by GTPase. Rheb has found to be expressed in prostate cancer and could promote tumourigenesis with PTEN haplo-insufficiency [16]. mTORC1 regulates autophagy by controlling protein synthesis through regulation of transcriptional regulators 4E binding protein 1 (4E-BP1) and p70 ribosomal protein S6 kinase (p70S6K) while mTORC2 is independent to nutrient availability and phosphorylates Akt and inhibits autophagy [4, 16]. mTORC1 activation would also inhibit autophagy by inhibiting Unc-51-like kinase 1 (ULK1) and the inhibition of mTORC1 could trigger autophagy and be used in cancer therapy. According to the name of mTOR, its target is rapamycin, which has been utilized for cancer therapy, such as its derivative RAD001 which is used in the treatment of renal cancer. Other mTOR inhibitors include pp242, Torin 1 and 2, temsirolimus, could be used to activate autophagy [2]. Apart from mTOR inhibitors, the negative regulator of the PI3K/Akt pathway AHRI, dual PI3K/mTOR inhibitors PI-103 and NVP-BEZ235, Akt inhibitors and the tumour suppressor gene PTEN may also be used to promote cell death in tumour cells [2, 4].

### UPR

**Fig. 3:**
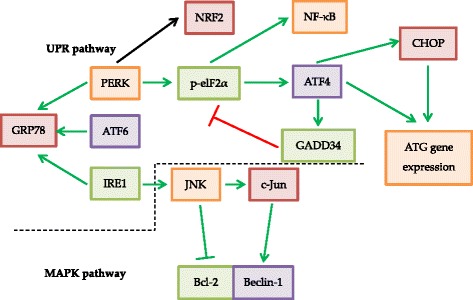
Title: For UPR signaling pathway, PERK and IRE1 activate autophagy through endoplasmatic reticulum stress. PERK promotes ATF4 and CHOP, which results in the promotion of ATG gene expression. IRE1 activates JNK, which belongs to the MAPK pathway. JNK promotes autophagy through the inhibition of Bcl-2 and activation of Beclin-1. Legend: Green arrows represent activating or repressing processes for autophagy stimulation and the red arrow represent a repressing process for autophagy inhibition

Apart from the PI3K-Akt-mTOR pathway, UPR (Fig. 3) might also regulate autophagy to relieve stress and re-establish cell homeostasis. Different pathological conditions from cancer and various cancer treatments, such as radiotherapy, could cause an accumulation of misfolded proteins in the ER and result in ER stress. Therefore, UPR may be up-regulated by some cancer types for improving tumour growth and therapy resistance. The pathway of UPR includes three parallel operating components, including PERK, activating transcription factor 6 (ATF6) and inositol requiring enzyme-1 (IRE1). These three components are affected by glucose-regulated protein 78 (GRP78), which is an ER chaperone and deactivates the three components by binding them in a normal condition. When there is an accumulation of unfolded proteins in the ER, GRP78 would bind to the unfolded proteins instead of the three components and thus activate the three components. Among the three components, the PERK-arm has been linked to radiation-induced autophagy for improving tumour cell survival under radiotherapy [14]. The PERK-arm contributes to hypoxia tolerance and phosphorylates elF2α, which leads to the general stop of protein synthesis in order to lessen the protein load in the ER. On the contrary, some cells may have a non-phosphorylatable eIF2α mutation and increase hypoxia sensitivity. Although transcription has been generally stopped, some factors are up-regulated such as NF-E2-related factor 2 (NRF2), nuclear factor κB (NF-κB) and activating transcription factor 4 (ATF4). NRF2 could induce transcription of cytoprotective genes under stress and provide resistance to anticancer therapies and aggressive tendency in cell proliferation with NRF2 accumulation leading to poor prognosis in non-small-cell lung cancer [17]. NF-κB activation causes the production of anti-apoptotic proteins [18], while ATF4 allows the restoration of normal ER function through the induction of C/EBP homologous protein (CHOP), growth arrest and DNA damage-inducible protein 34 (GADD34) and lysosome-associated membrane protein 3 (LAMP3). CHOP is the pro-apoptotic component of the UPR and mediates cell death when the cell adaptation fails to withstand the ER stress whereas GADD34 is able to dephosphorylate eIF2α as a negative feedback loop. LAMP3 induces fusion of autophagosome and lysosome, which increases resistance to cancer therapies and promotes metastasis [2].

### Mitogen-activated protein kinases (MAPK)

MAPK (Fig. 3) regulate cell proliferation and survival by autophagy, which includes c-Jun N-terminal kinase (JNK) and extracellular signal-regulated kinase (ERK). JNK is activated by various stresses and it mediates autophagy both directly and indirectly. Directly, JNK can promote cell death in cancer cells by inducing p53 and Atg5. Indirectly, JNK inhibits the association of Bcl-2 with Beclin-1 and upregulates Beclin-1 expression by c-Jun phosphorylation. JNK could be inhibited by SP600125 which in turn inhibits Beclin-1 expression and autophagy [2]. Whereas ERK is activated by active cell proliferation signals and significantly overexpressed in cancer cells. ERK is a component of the proto-oncogene c-RAF (Raf)- mitogen-activated protein kinase kinase (MEK)-ERK pathway of the Ras small GTPase. The Ras family has frequent mutations in cancer cells, which creates a high level of basal autophagy in cancer cells even if there is a good supply of nutrients. Ras is an up-regulator of Raf-MEK-ERK pathway by binding and activating Raf. Raf then activates MEK and MEK can activate and phosphorylate ERK1 (p44) and ERK2 (p42), which can promote autophagy without other stimuli. Apart from promoting Raf-MEK-ERK pathway, Ras can also activate the PI3K pathway for repressing autophagy and thus Ras has a regulatory role in autophagy [4].

## Effect of autophagy modulation on radiotherapy efficacy in different cancer types

Since autophagy can sustainably affect cell proliferation and survival, various trials have been conducted to modulate autophagy for improving the outcome of cancer treatment in combination with currently used treatment modalities such as radiotherapy in different cancer types. Table 1 summarises the effects of autophagy modification in improving radiosensitivity or radiotherapy efficacy through various autophagy signalling pathways in different cancer types.

**Table 1:** Effects of autophagy modifications on improving radiosensitivity or radiotherapy efficacy

Cancer type	Cell line	Autophagy agent (Induction (+)/ Inhibition (−))	Autophagy pathway affected	Animal study (Yes (+)/ No (−))	Reference
Glioblastoma	T98G + U373MG	Rapamycin (+)	PI3K-Akt-mTOR (mTOR inhibitor)	-	Palumbo et al. [19]
	SU2	NVP-BEZ235 (+)	PI3K-Akt-mTOR (PI3K/mTOR inhibitor)	-	Wang et al. [20]
	U373MG	Chloroquine (−)	UPR (PERK)	+	Rouschop et al. [21]
Oral cancer	OC3 + SAS	Rapamycin (+)	PI3K-Akt-mTOR (mTOR inhibitor)	-	Wu et al. [24]
Lung cancer	H460	RAD001 (+)	PI3K-Akt-mTOR (mTOR inhibitor)	+	Kim et al. [25]
	H460	Rapamycin (+)	PI3K-Akt-mTOR (mTOR inhibitor)	+	Kim et al. [26]
	CDDP-Resistant H460	NVP-BEZ235 (+)	PI3K-Akt-mTOR (PI3K/mTOR inhibitor)	-	Kim et al. [27]
Breast cancer	MDA-MB-23 + MCF-7	RAD001 (+)	PI3K-Akt-mTOR (mTOR inhibitor)	-	Albert et al. [29]
	MCF-7	Rapamycin (+)	PI3K-Akt-mTOR (mTOR inhibitor)	-	Paglin et al. [13]
Oesophageal cancer	EC109	Tunicamycin (+)	PI3K-Akt-mTOR (ER stressor)	+	Pang et al. [30]
Pancreatic cancer	MIA PaCa-2 + PANC-1	MG132 (+)	MAPK (JNK) (Proteasome inhibitor/ ER stressor)	+	Chiu et al. [32]
Colorectal cancer	HCT-116	BCG/CWS (+)	MAPK (JNK/ERK)	+	Yuk et al. [33]
	HCT-116 + HT-29	Chloroquine (−)	UPR (PERK)	+	Rouschop et al. [21]
Prostate cancer	Biopsy specimens	MG132 (+)	MAPK (JNK) (Proteasome inhibitor/ ER stressor)	-	Koukourakis et al. [34]
	DU145 + PC3	RAD001 (+)	PI3K-Akt-mTOR (mTOR inhibitor)	-	Cao et al. [35]

### Glioblastoma

Glioblastoma is an aggressive brain cancer with a poor prognosis despite the use of a multimodality treatment such as radiotherapy combined with Temozolomide (TMZ) [19]. Past studies have been performed to investigate the possible adjuvant therapy by controlling cell death. In an in vitro study, the difference between high radiosensitivity glioblastoma cell line T98G and low radiosensitivity cell line U373MG in autophagy has been studied and it was found that autophagy activation after radiotherapy was more prominent in T98G than U373MG from the assessment of Beclin-1 and Atg5 expressions. After adding the mTOR inhibitor rapamycin in the two cell lines, autophagy inductions were observed and radiosensitivities were enhanced in both cell lines [19]. A similar result was obtained by Wang et al. (2013) [20], who used a dual PI3K/mTOR inhibitor NVP-BEZ235 in glioblastoma cell line SU2 and demonstrated that NVP-BEZ235 could radiosensitise SU2 by activating autophagy. While another study focussed on UPR and hypoxia, the results showed that hypoxia was able to stimulate autophagy in U373MG cells from increased expression of MAP1LC3B and Atg5 [21]. MAP1L3B is activated by transferring from cytosolic MAP1LC3B-I to lapidated membrane-bound form of MAP1LC3B-II, and this is strictly dependent on Atg5. Also, these two autophagy genes are regulated by the PERK-arm of UPR and PERK is able to activate elF2α, which in turn activates ATF4 and CHOP. Further analyses in the study showed that ATF4 and CHOP activate MAP1LC3B and Atg5 respectively. The adding of the lysosomotrophic agent chloroquine (CQ) blocked the final step of autophagy, causing further accumulation of MAP1LC3B apart from hypoxic stress and this addition of CQ has been found to increase the radiosensitivity of xenograft from U373MG cells [21]. The positive results from these studies may be considered for the development of new treatment strategies and improve the prognosis of this deadly cancer type.

### Oral cancer

Oral cancer comprises about 85% of all head and neck cancers and the 5-year survival has shown little improvement for decades [22, 23]. The main treatment modalities include surgery and radiotherapy and squamous cell carcinoma is the most common neoplasia of oral cancer. Thus, Wu et al. (2014) [24] investigated the relationship between radiation and autophagy in human oral squamous cell carcinoma cell lines OC3 (Derived from betel quid chewing patients) and SAS (Derived from non-betel quid chewing patients) in an in vitro experiment. The results showed irradiation was able to induce autophagy in both OC3 and SAS cells with OC3 having a greater increase in the rate of autophagy. Further analysis showed the involvement of the mTOR pathway in both cell lines but the upstream autophagy pathways involved were different for the two cell lines. Autophagic degradation occurred in irradiated OC3 cells while autophagosome accumulation occurred in irradiatied SAS cells. Therefore, irradiation-mediated cell death occurred only in OC3 cells and irradiation did not reduce cell viability in SAS cells. Treatment of rapamycin (autophagy inducer) in combination with irradiation in OC3 cells resulted in a further reduction in cell viability, suggesting the synergistic effects of irradiation and autophagy incuction in OC3 cells [24]. The difference in irradiation-induced growth inhibition between the two cell lines requires further investigation in order to decipher the actual mechanisms of irradiation-mediated autophagic degradation.

### Lung cancer

As lung cancer is one of the most common cancers and current treatment modalities of radiotherapy and chemotherapy are only able to yield moderate survival benefits with significant side-effects. Research has been conducted on autophagy to improve the treatment outcome, especially for advanced non-small cell lung cancer (NSCLC) [25]. The main focus is mTOR inhibition combined with apoptosis induction or inhibition. In a study conducted by Kim et al. (2008) [25], mTOR inhibitor (autophagy inducer) RAD001 and caspase-3 inhibitor (apoptosis inhibitor) Z-DEVD were injected into H460 lung cancer xenografts in vivo. The results showed that the combined use of RAD001 and Z-DEVD followed by radiotherapy treatment caused the greatest delay in tumour growth, for 9 days, when compared with radiotherapy alone. Also, there were the greatest reductions in cellular proliferation by Ki67 staining and angiogenesis by von Willebrand Factor (vWF) staining in the tri-modality treatment group. Moreover, in a further research study by Kim et al. (2009) [26], the combined use of mTOR inhibitor (rapamycin) and apoptosis inducer (Bcl-2 inhibitor) ABT-737 with radiotherapy led to further tumour control in the H460 lung cancer mouse xenograft model. Their results include increase in tumour growth delay by 7 days, decrease in cell proliferation by 77% and vascular density by 67.5% when compared with radiotherapy alone [26]. Another study demonstrated the enhancement of radiosensivity by using NVP-BEZ235 to block the PI3K/mTOR pathway in cisplatin-resistant NSCLC tumour cells both in vitro (reduced survival fraction after irradiation) and in vivo (increase in tumour growth delay) settings [27]. These research studies show the importance of autophagy in the control of lung cancer through autophagy mechanism. Combined with irradiation and autophagy promotion, the first two studies demostrated the most prominent cell death effect during apoptosis inhibition and promotion respectively [25, 26]. This conflicting results warrant further research in respect of the interaction between autophagy and apoptosis, particularly for the quantification of both autophagy and apoptosis levels.

### Breast cancer

The relationship between irradiation and autophagy was also studied in breast cancer cells. In an in vitro study, radiotherapy was found to induce autophagy in the MDA-MB-23 breast cancer cell line through PI3K-Akt-mTOR pathway, in which the rate of autophagy was increased to improve tumour cell survival [28]. Another study also confirmed the role of autophagy in MCF-7 and MDA-MB-231 breast cancer cell lines. The addition of mTOR inhibitor RAD001 promotes autophagy and increases the radiosensitivity of tumour cells in an in vitro model [29]. The action of mTOR inhibitor in radiotherapy treatment of breast cancer was further studied by Paglin et al. (2005) [13]. In addition to increased cell death, the combination of autophagy induction and radiotherapy showed an increase in mitochondria hyperpolarization. The results were confirmed by the decrease in mitochondrial membrane potential, signalling mitochondria dysfunction and increase in phosphorylation of p53 at Ser15, which is a regulator of apoptosis and tumour suppressor mutated in 50% of all tumours [13]. These results showed that autophagy might help breast tumour cells to survive under treatment stress and the suitable control of autophagy might improve the treatment outcome of radiotherapy.

### Oesophageal cancer

Oesophageal cancer is also a cancer with a poor prognosis which is treated by radiotherapy combined with other treatments. However, different tumours of the same grade have differential radiosensitivities and this limits the effectiveness of radiotherapy. Thus, a recent study investigated ER stress and autophagy in oesophageal cancer. An ER stress inducer, TM was applied to the oesophageal cancer cell line EC109 followed by irradiation [30]. An increase in acute cell death and decrease in colony survival were observed, showing the radiosensitising property of TM. Also, enhanced apoptosis resulting from an increase in cleaved caspase-3, and increased autophagy from increased LC3-I/LC3-II ratio in Western blotting analysis were noted. Moreover, mTORC1 was significantly up-regulated and PI3K and phosphorylated Akt reduced after TM treatment, suggesting the involvement of PI3K-Akt-mTOR pathway in radiosensitising EC109 cells. However, the inhibition of autophagy by Beclin-1 knockdown showed an increase in apoptosis and decrease in cell viability, thus autophagy could also have cytoprotective roles in stressed tumour cells [30]. The experiment was repeated using a mouse model and the observation of delay in tumour growth and involvement of PI3K-Akt-mTOR pathway validated the in vitro findings. The findings are controversial as autophagy causes both cell death or provides cytoprotection, which could be due to the influence of tumour stages on the character of autophagy as autophagy usually acts as a tumour suppressor in the early stages and provides tumour protective functions in developed tumours [31].

### Pancreatic cancer

Pancreatic cancer also has a poor prognosis under current combined treatment. Chiu et al. (2015) [32] investigated the action of a proteasome inhibitor MG132 and radiotherapy in two pancreatic cancer cell lines MIA PaCa-2 and PANC-1 in vitro and in vivo. Results showed that the combined modalities increased ER stress, increased IRE1α protein level, promoted autophagy induction, increased detection of early apoptosis and cell death and increased MAP1LC3 level. The results were cross validated by adding the autophagy inhibitor 3-methyladenine (3-MA). The experiment was also repeated with an animal model and it was found that the combined treatment was able to increase tumour growth delay when compared with the MG132 or radiotherapy alone group [32]. This study also showed the down-regulation of TRAF6 though the combination of MG132 and irradiation. As TRAF6 represents a potential target for tumourigenesis in pancreatic cancer, further investigation is suggested for employing autophagy modulation in improving prognosis.

### Colorectal cancer

Colorectal cancer is the third most common cancer in men and the second most common cancer in women worldwide with surgery as the primary treatment. Whereas radiotherapy is widely used for rectal cancer as preoperative or postoperative adjuvant therapy [22]. Efforts have been made to investigate the modification of autophagy in improving treatment effectiveness. Yuk et al. (2010) [33] used Mycobacterium bovis Bacillus Calmette-Guerin (BCG/CWS) as an inducer of cell death in both in vitro and in vivo models. It is found that BCG/CWS could radiosensitise HCT-116 colorectal cancer cells via autophagy. Further investigation of the signalling pathway showed the generation of ROS due to up-regulation of JNK and ERK. Thus, the MAPK pathway was involved in the autophagy process. The efficacy of radiotherapy treatment of HCT-116 tumours in the mouse model was found to be significantly improved when combined with BCG/CWS [33]. Another study done by Rouschop et al. (2010) [21] focussed on hypoxia and the effect of autophagy in radiosensitivity. Their results showed that hypoxia could stimulate autophagy in HCT-116 and HT-29 colorectal cell lines and decreased radiosensitivities in vitro. Also, MAP1LC3B and Atg5 were found to be involved and regulated by the PERK-arm of UPR. Inhibition of autophagy by adding CQ caused the increase of radiosensitivity. Verification of experimental results was done in xenograft of HCT-116 tumour in mouse and the use of CQ in blocking autophagy and increasing radiotherapy efficacy was validated [21]. Although the two studies employed different strategies in autophagy modulation for improving radiosensitivity, both measures tried to perturb autophagy from basal levels to improve treatment results.

### Prostate cancer

Prostate cancer is a common cancer in men and is usually treated by radiotherapy and radical prostatectomy. Although the current treatment has a high therapeutic efficacy, relapses are common among high risk patients and thus radiosensitisation is studied to improve treatment effectiveness [34]. In a study comparing 54 biopsy specimens of prostate cancer with normal prostate tissue, it was found that prostate cancer cells had a higher level of light chain 3A (LC3A) and lower level of lysosome-associated membrane protein 2 (LAMP2a), which are markers of autophagosome and lysosome cellular content respectively. Moreover, the study used two cell lines DU145 (radiosensitive) and PC3 (radioresistant) to study the level of autophagy after irradiation. Results showed that PC3 has a greater autophagic flux after irradiation than DU145 with a higher level of LC3A and a lower level of LAMP2a. Adding MG132 (blocker of proteasome pathway) caused a decrease in LC3A and an increase in p62 and LAMP2a, showing the halt of autophagy and this radiosensitised both cell lines. Therefore, this study concluded that a high level of autophagy could compromise the treatment efficiency of radiotherapy [34]. Another study investigated the use of mTOR inhibitor RAD001 for radiosensitising prostate cancer cell lines DU145 and PC3. Also, the additional use of Z-VAD, the inhibitor of caspase-dependent apoptosis, was found to further enhance autophagy and the cytotoxic effects of radiation. Finally, PTEN (common mutation of the suppressor of PI3K-Akt pathway) deficient cell lines could offer some further improvement of radiosensitisation. Thus, the promotion of autophagy by mTOR inhibition may improve radiotherapy efficacy in prostate cancer [35]. These two studies provided opposing views on the promotion of autophagy to improve the radiosensitivities of prostate cancer cells. The discrepancy could be due to the different autophagy levels attained by different autophagy promoters. Thus, accurate quantification of autophagy level should be developed for understanding the influence of autophagy in prostate cancer treatment.

## Summary of autophagy modulation on improving radiotherapy efficacy in different cancer types

The strategies used in the major studies on different cancer types can be divided into three main categories. The most common direction is the induction of autophagy through promoting the PI3K-Akt-mTOR pathway, mainly by using mTOR inhibitors. This strategy has been adopted among glioblastoma, oral, lung, breast, oesophageal and prostate cancers. The second direction is the induction of autophagy through promoting the MAPK pathway by inducing ER stress. This has been employed in pancreatic, colorectal and prostate cancers. The third category is the inhibition of autophagy through the UPR pathway, which is achieved by the addition of chloroquine as exemplified in the glioblastoma and colorectal cancer studies. All strategies are aimed at adjusting the autophagy level of tumour cells to improve treatment efficacy.

## Future development

As summarised above, there were conflicting results regarding the effect of autophagy in radiosensitising tumours for improving radiotherapy efficacy. Therefore, more investigation is needed before implementing modulation of autophagy in clinical settings [36]. There are two aspects which have been identified for further development. The first aspect is the establishment of autophagy level for individual tumours. It is difficult as autophagy is hard to quantify, for example, the number of autophagosomes is not directly related to the level of autophagy as an increased number of autophagosomes could represent a reduced rate of turnover apart from an increased rate, and a reduced number of autophagosomes may represent an increase of autophagic flux [2]. Moreover, tissue biopsy experiments would have to be performed for individual patients instead of relying on established cancer cell line results because different tumour samples may have varied basal autophagy levels and tumour microenvironments. Also, personalised treatments by modifications of tumour microenvironment and treatment modalities schemes would need to be investigated. In particular, hypoxia and the varied radiotherapy dose scheme, and differences in autophagy pathways between preclinical and clinical studies need to be investigated as the cellular pathways have multiple feedback loops and backup mechanisms. This way, the full picture of autophagy mechanism may be obtained and its relationship with current cancer treatment modalities such as radiotherapy may be used with higher precision. The second aspect is the specialisation of autophagy induction or inhibition drugs. Currently, autophagy inducers or inhibitors used in preclinical studies mostly have different primary usages, such as CQ, which is a drug used for the treatment of malaria [2]. These drugs have systemic effects and are not specifically targeted at tumour cells. It is possible that the radiosensitivity of normal tissue is also increased, leading to an increase in radiation injuries [37]. Moreover, the mTOR inhibitor rapamycin has been used in early clinical trials and was found to cause proteinuria as podocytes depend on the mTOR pathway to control autophagic flux for proper functioning [38]. Therefore, specialised autophagy induction or inhibition agents with tumour specific uptake and accumulation are needed to enable modification of autophagy and reducing side effects at the clinical stage.

## Conclusion

Autophagy, a vitally important cellular mechanism that affects tumour survival and proliferation, provides a valuable target for enhancing cancer treatment efficacy apart from current modalities such as radiotherapy. However, as autophagy is a two-edged sword that may promote the destruction or protection oftumour cells, based on the different tumour type and stage, and may interact with current treatment modalities, resulting in an improved or worsened prognosis. More extensive research and development is needed to successfully modify autophagy for clinical gains, especially for establishing a quantified autophagy level with an individual tumour microenvironment and treatment scheme considerations, and the specialisation of autophagy agents for overcoming the systemic effect of autophagy agents on patients.

## Abbreviations

3-MA: 3-methyladenine
4E-BP1: 4E binding protein 1
Akt: Protein kinase B
ATF4: Activating transcription factor 4
ATF6: Activating transcription factor 6
ATG: Autophagy gene
BCG/CWS: Mycobacterium bovis Bacillus Calmette-Guerin
Bif-1: Bax interacting factor 1
CHOP: C/EBP homologous protein
CQ: Chloroquine
eIF2α: Eukaryotic initiation factor 2α
ER: Endoplasmic reticulum
ERK: Extracellular signal-regulated kinase
ERp29: Endoplasmic reticulum protein 29
GADD34: Growth arrest and DNA damage-inducible protein 34
GBM: Glioblastoma Multiforme
GRP78: Glucose-regulated protein 78
IRE1: Inositol requiring enzyme-1
JNK: c-Jun N-terminal kinase
LAMP2a: Lysosome-associated membrane protein 2
LAMP3: Lysosome-associated membrane protein 3
LC3A: Light chain 3A
MAP1LC3: Microtubule-associated protein 1 light chain 3β
MAPK: Mitogen-activated protein kinases
MEK: Mitogen-activated protein kinase kinase
mTOR: Mechanistic target of rapamycin
mTORC1: Mechanistic target of rapamycin complex 1
mTORC2: Mechanistic target of rapamycin complex 2
NF-κB: Nuclear factor κB
NRF2: NF-E2-related factor 2
NSCLC: Non-small cell lung cancer
p70S6K: p70 ribosomal protein S6 kinase
PERK: Protein kinase-like endoplasmic reticulum kinase
PI3K: Phosphatidylinositol-3-kinase
Raf: Proto-oncogene c-RAF
Rheb-GTP: Ras homolog enriched in brain
ROS: Reactive oxygen species
RPS6KB1: Ribosomal protein S6 kinase
TM: Tunicamycin
TMZ: Temozolomide
TSC: Tuberous sclerosis complex
ULK1: Unc-51-like kinase 1
UPR: Unfolded protein response
UVRAG: UV radiation resistance-associated gene
vWF: Von Willebrand Factor
